# Analysis of an Imported Subgenotype C2 Strain of Human Enterovirus 71 in Beijing, China, 2015

**DOI:** 10.3389/fmicb.2018.02337

**Published:** 2018-09-28

**Authors:** Jie Li, Yindong Li, Songjian Zhang, Hongmei Ma, XiaoXiao Liu, Zhichao Liang, Wenzeng Zhang, Hongbo Jing, Yiwei Du, Yang Yang, Da Huo, Lijuan Chen, Quanyi Wang

**Affiliations:** ^1^Beijing Center for Disease Prevention and Control, Beijing, China; ^2^Beijing Center for Preventive Medicine Research, Beijing, China; ^3^Beijing Shunyi Center for Disease Prevention and Control, Beijing, China; ^4^Beijing Xi Cheng Center for Disease Prevention and Control, Beijing, China

**Keywords:** human enterovirus 71, subgenotype C2, neutralizing antibody, cross-protectivity, Antigenic variation

## Abstract

**Background:** Subgenotype C4 of enterovirus 71 (EV71) is the predominant agent of Hand Foot and Mouth disease (HFMD) circulating in the mainland of China. For the first time, a subgenotype C2 of EV71 named SY30-2 was isolated from a HFMD case in Beijing, China. Since it is uncertain whether antibodies raised against subgenotype C4 of EV71 can protect C2 EV71, it is important to monitor and check the presence of cross-reactive antibodies against new EV71 subgenotypes. To find out the causes for the different NtAb, this study is to investigate the relationships between amino acid residue variations and cross-reactive antibodies against EV71 subgenotypes C2 and C4.

**Methods:** Nucleotide and amino acid sequences from full-length genome sequence of SY30-2 were compared to EV71 reference strains. A microneutralization test was used to detect neutralizing antibody (NTAb) in the sera of subgenotype C4 of EV71 infected cases against SY30-2 and FY17 (a C4 isolate). The 3D structure of the viral capsid protein of SY30-2 was constructed.

**Results:** Genome sequence and similarity plot analyses showed that SY30-2 shared the highest identity with subgenotype C2 of EV71 strains in every fragment of the genome. While the microneutralization test result showed that children infected with subgenotype C4 of EV71 had higher NTAb titers against FY17 than SY30-2 (*p* < 0.001). The amino acid sequence comparison revealed that four amino acid residues VP1-22, VP1-31, VP1-249 and VP3-93 were highly conserved in subgenotype C4 of EV71 compared with the corresponding amino acid residues on subgenotype C2 of EV71 (*p* < 0.05). Furthermore, the 3D-structure of viral capsid protein showed that VP1-22, VP1-31 and VP3-93 were located on the surface of virion.

**Conclusion:** This is the first report of an EV71 subgenotype C2 isolated from HFMD in Beijing, China. Only a few antigenic variations on subgenotype C2 of EV71 could have led to a great decrease in NTAb titer. Thus, imported new genotypes and subgenotypes of EV71 should be closely monitored. The efficacy of available vaccines against new viruses should be evaluated as well.

## Introduction

Hand Foot and Mouth Disease (HFMD) is a very common infectious disease usually associated with children younger than 5 years old. EV71 is a member of the Enterovirus genus of the *Picornaviridae* family (Xing et al., 2014). It is one of the major causative pathogens of HFMD and the most common etiological agent isolated from HFMD patients complicated with neurological disorders in China.

The EV71 particle has a non-enveloped, icosahedral capsid comprising 60 protomers. Each protomer consists of four viral proteins VP1, VP2, VP3 and VP4 (Ranganathan et al., 2002). VP1, VP2, and VP3 are on the surface of the virion and VP4 is arranged internally. The VP1, VP2, and VP3 proteins are exposed on the virion surface and are responsible for host-receptor binding and immune responses (Brown and Pallansch, 1995; Chen et al., 2010). EV71 is currently classified into 3 genotypes, A, B and C and the genotypes B and C are further divided into subgenotypes B1–B5 and C1–C5 (Zhang et al., 2009; Solomon et al., 2010). Recently, genotype D, E and F were detected (Deshpande et al., 2003; Bessaud et al., 2014). Since the first reported detection of EV71 in 1998, the subgenotype C4 has been the predominant agent circulating in the mainland of China. (Yan et al., 2012). While in Asia-Pacific region, there are a variety of genotypes EV71. (World Health Organization [WHO], 2011). There is a great possibility of importing other genotypes EV71 into China. Subgenotype C2 of EV71 has been prevalent in many countries for several years (World Health Organization [WHO], 2011). The first subgenotype C2 of EV71 in China was reported in 2012 and was obtained from an acute flaccid paralysis (AFP) in 1996 (Tao et al., 2012). It showed high identity with C2 reference strains in VP1 gene. It has been reported that antibody responses against subgenotype C4 of EV71 can cross protect infections of other subgenotypes (Huang et al., 2010). However, according to a previous study, a C2-like strain which was genetically recombinant couldn’t be effectively neutralized by serum from C4 infected cases (Huang et al., 2013). Till now, the relationship between the antibody cross-protectivity and antigenic properties of these strains remained unknown.

In this current study, based on the identification of a EV71 subgenotype C2, we attempted to combine the result of NTAb titer against subgenotypes C4 and C2 of EV71 with corresponding viral genetic sequence data to identify relationships between cross-reactive antibodies and amino acid residue variations. Understanding of the antigenicity of diverse EV71 isolates is crucial for the surveillance strategy formulation, especially after EV71 vaccine is widely used in China.

## Materials and Methods

### Sample Source

In this study, the SY30-2 strain was isolated from a boy of 3 years of age. He traveled to Thailand with his parents from April 5 to 11 in 2015. On April 11, he got the symptom of fever and rash on hands and feet, and had no neurological complications. On April 13, his parents took him to see a doctor. His throat swab and information were collected by pediatrician after obtaining the informed consent of the parents. The throat samples were kept in minimum essential media (MEM) and stored at 4°C until transfer to Beijing CDC for laboratory test. FY17 is a subgenotype C4 of EV71 strain isolated in FuYang, China in 2008. It was obtained from China CDC.

Non-anticoagulated whole blood and information were collected from the index case of each HFMD outbreak in Beijing after obtaining the informed consent of the guardians. The blood samples were first stored in a 4°C refrigerator overnight. The next morning, the serum of each sample was dispensed into serum preservation tubes and stored at -20°C.

### Nucleotide Extraction, Virus Identification and Enterovirus Isolation

Total nucleotide extraction was carried out with a Roche MagNA Pure LC 2.0 nucleic extraction system (ROCHE, Co, United States) using MagNA Pure LC Total Nucleic Acid Isolation Kit–Large Volume (ROCHE, Co, United States), according to the manufacturer’s instructions.

Complete nucleotide sequences of the VP1 gene were amplified using specific primers as previously described (Mirand et al., 2010). PCR products of completeVP1 genes were purified and sequenced using ABI PRISM 310 Genetic Analyzer.

Human rhabdomyosarcoma (RD) cells were used to isolate enterovirus from the supernatant of the throat swab specimen. The RD cells were cultured using MEM with 10% fetal bovine serum till 75% of flask bottom was covered by monolayer RD cell. Then the cell culture medium was removed and the supernatant of the specimen was inoculated on the RD cells. After 1 h of incubation at 36°C, the specimen supernatant was replaced by MEM with 2% fetal bovine serum. Cytopathic effect of the enterovirus infected RD cells was observed every day. Cells were collected when 75–90% covered cells showed cytopathic effect.

### Determination of the Full-Length Genome Sequence of EV71

The full-length genome of EV71 was amplified from the throat sample with the primers of SY30-2-F (5′-TTA AAA CAG CCT GTG GGT TGC-3′), SY30-2-R3296 (5′-TGG ATT GGC TTT GAA TAG ATA-3′), SY30-2-F3036 (5′-CCC ACA TTC GGT GAA CAC AAG C-3′) and SY30-2R-3′ (TTT TTT TTT TTT TTT TTT TTG CTA TTC TGG). The reverse transcription reaction was performed at 42°C for 1h with reverse transcriptase (PrimeScript^TM^ 1st strand cDNA Synthesis Kit, Takara) using the RNA as the template and the primer SY30-2R-3′ as the specific reverse transcription primer. A DNA fragment containing approximately 3 kb of the 5′region of the viral genome was amplified with primers SY30-2F-5′ and SY30-2-R3296-3′. The DNA fragment containing approximately 4 kb of 3′ region of SY30-2 was amplified by PCR with primers SY30-2-F3036-5′ and SY30-2R-3′. Both PCRs were performed under the following conditions: initial denaturation for 1min at 94°C; 33 cycles of 30s at 94°C, 30s at 60°C and 8 min at 72°C; with a final extension of 72°C for 10 min. Obtained PCR products were sequenced with SY30-2F-5′ and SY30-2R-3′, respectively. Based on the obtained sequence, new primers were designed and PCR products were sequenced until the end of the product.

### Sequence Analysis and Recombination Analysis

Identification and subtyping was carried out by sequence comparisons with reference sequences in GenBank using the BLAST and confirmed by phylogenetic analysis. The nucleotide sequences of EV71 strains were aligned with reference sequences using the Clustal W tool within BioEdit software (version 5.0). Bayesian Markov chain Monte Carlo (BMCMC) methods were used to analyze the phylogenesis of the VP1 genes of EV71 using BEAST v1.8. Bayesian analyses were performed using a relaxed molecular clock model. Phylogenetic trees were displayed and annotated by using FigTree. Pairwise distances of nucleotide and amino acid sequences between different gene fragments were calculated with MEGA software (version6). Similarity plot analysis was performed using the Simplot program (version 3.5.1). A sliding window of 200 nucleotides was used, moving in 20 nucleotide steps.

### Determination of Virus Titers, NTAb Assay

Virus titer was determined as the median end-point of the tissue culture infectious dose (TCID50) by a microtitration assay using a standard protocol (Bey and Golombick, 1984). NTAb against FY17 and SY30-2 were detected with a neutralization test by micro technique on RD cell line, as previously described (Zhu et al., 2010). All serum sample determinations were performed in duplicate. The serum samples were first inactivated at 56°C for 30 min, and then 50uL of four-fold serially diluted sera (10 serial dilutions were prepared) and 50uL of virus working solution containing 100 TCID50 of EV71 were mixed on 96-well microplates and incubated with 100ul RD cells.

### Properties of Amino Acid Residues

The amino acid property classes were defined for the hydropathy, volume and chemical characteristics. According to the Kyte and Doolittle amino acid hydropathy index (Kyte and Doolittle, 1982), three ‘hydropathy’ classes were defined: hydrophobic (I, V, L, F, C, M, A, W), neutral (G, T, S, Y, P, H) and hydrophilic (D, N, E, Q, K, R). Amino acid volumes in angstrom were used to define the amino acid ‘volume’ classes. Five classes were defined: very small (G, A. S), small (C, D, P, N, T), medium (E, V, Q, H), large (M, I, L, K, R) and very large (F, Y, W). Based on the principal chemical property of the amino acid side chain (Pommie et al., 2004), eleven amino acid ‘chemical characteristics’ classes were defined: aliphatic (A,V, I, L); sulfur (C, M); hydroxyl (S, T); acidic (D, E); amide(N, Q); basic (H, K, R) and five classes correspond to a single amino acid (F, W, Y, G, P) owing to their particular characteristics.

### Labeling Amino Acid Sites on the 3D Structure of EV71 Capsid Protein

The 3D structure of the EV71 capsid protein was constructed using SWISS-MODEL by homology modeling. The crystal structure of EV71 (PDB: 4CEW) was used as template for the model. Specific amino acid residues were located using the Pymol software.

### Statistical Analysis

Statistical analysis was conducted by SPSS19 software (IBM SPSS Inc., Chicago, IL, United States). NTAb titers were log transformed to calculate the geometric mean titers (GMTs). Undetectable titer was assigned a level of 0 for the calculation of GMT. Paired *t*-test or Wilcoxon signed rank test were used to compare the difference in the NTAb between FY17 and SY30-2. Pearson’s χ^2^ test was used to compare the difference of constituent ratio of amino acid residue between subgenotypes C2 and C4. Odd Ratio (OR) was calculated to determine the association between the subgenotype and amino acid residue. A *p*-value of < 0.05 (2-sided significance testing) was considered statistically significant in above analyses.

### Ethics Statement

This study was carried out in accordance with the recommendations of institutional review board and human research ethics committee of the Beijing CDC. The approval number of the local ethics committee was 201702. Samples (throat swabs and serum specimens) collection in this study was agreed by the patient’s guardian with prior written informed consent which was in accordance with the Declaration of Helsinki. The protocols were approved by the institutional review board and human research ethics committee of the Beijing CDC.

## Results

### Genotype and Subgenotype Identification, Genome Recombination Analysis

**FIGURE 1:**
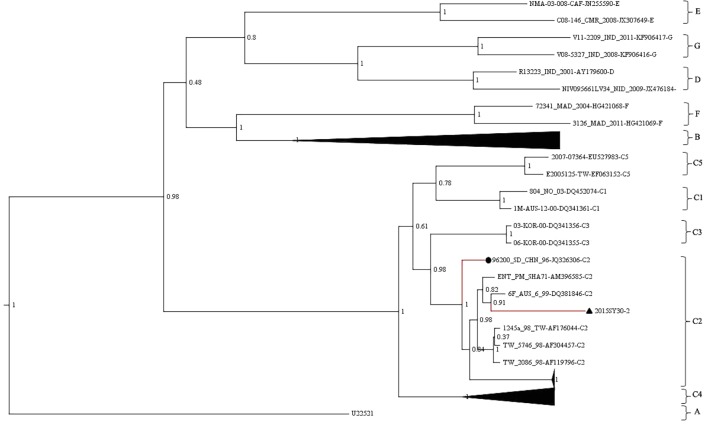
Phylogenetic tree based on the entire VP1 coding region (891bp) of EV71. The analysis was done using BEAST v1.8. The posterior probability was used to determine the node support. Black triangle indicates the first subgenotype C2 of EV71 (SY30-2) isolated from a HFMD case in China. Black dot indicates the first subgenotype C2 of EV71 (JK326306) isolated from an AFP case isolated in China.

The whole VP1 sequence (891bp) of SY30-2 was obtained and aligned with EV71 reference strains including genotype A, B0-B7, C1-C5, D, E, F strains (GenBank accession numbers were listed in the phylogenetic tree). The isolates of each genotype and subgenotype clustered together on the phylogenetic tree based on the VP1 whole sequence. According to the phylogenetic tree and the nucleotide identity between SY30-2 and C2 reference strains (92.0–94.2%, calculated by the Kimura 2-parameter model, C2 reference strains JQ326306, AF304457, AF176044, AF119796, AM396585, DQ381846 were used), SY30-2 belonged to subgenotype C2 (**Figure 1**). The nucleotide and amino acid identity of the VP1 sequence between SY30-2 and a former isolated EV71subgenotype C2 (JQ326306) in China were 92.0 and 99.0%, respectively. The nucleotide and amino acid identities of the VP1 sequences between SY30-2 (MG214681) and C4 reference strains (GenBank accession number: AY465356, DQ133459, FJ606447, FJ713137, and FJ607334) were 85.8–87.6% and 98.6–99.0%, respectively. The phylogenetic placement of the SY30-2 strain was further confirmed by sequencing the whole genome and calculating the evolutionary distance with other subgenotypes of EV71. Results showed that SY30-2 genome sequence (GenBank accession number: MG214681) shared 91.6–92.9% nucleotide identity with subgenotype C2 reference strains (DQ381846, AF176044). The highest identity in each gene fragment was observed between SY30-2 and C2 reference strains compared with other subgenotypes of EV71 (**Table 1**). Similarity plot analyses result showed no recombination for SY30-2 isolate when compared with other subgenotype C2 of EV71 (**Supplementary Figure S1**).

**Table 1:** The percent identity (%) of nucleotide and amino acid sequences in different gene fragments between SY30-2 and other subgenotype of EV71.

		Subgenotype^∗^										
Gene		A	B1	B2	B3	B4	B5	C1	C2	C3	C4	C5
VP4	nt	80.5	79.4	76.7–77.5	78.2–78.9	79.6	79.0–81.1	82.4–83.7	88.8–91.7	87.7	81.1–83.1	82.5–83.8
	aa	100	98.5–100	97.1–100	100.0	100.0	100.0	98.5	100.0	100.0	97.1–100.0	100.0
VP2	nt	76.7	82.7–82.9	81.7–82.4	80.0–80.2	79.4–79.6	78.9–79.6	86–86.2	93.5–94.6	89.3–89.6	86.2–88.5	85.9
	aa	97.2	97.2	97.2–97.6	97.2–97.6	97.6	96.8–97.6	98.8–99.2	99.2–99.6	99.2	97.6–100.0	99.2
VP3	nt	78.2	77.3–77.5	77.5–78.1	76.3–76.4	74.9–0.75.2	77.5–78.3	85.6–85.9	90.7–91.3	87.0–87.4	87.2–87.9	83.7–84.1
	aa	97.9	97.5	97.1–97.5	96.6	97.1	97.1	99.2–99.6	100.0	100	99.2–99.8	100.0
VP1	nt	77.9	78.2–78.5	78.0–78.3	78.0	77.2–77.3	77.5–79.3	87.7–87.0	92.8–94.1	88.5–88.6	85.8–87.6	85.2
	aa	95.2	96.2–96.6	96.6	96.2	96.9	97.3–97.6	99.0	99.3–100	99.0	98.6–99.0	98.0–99.0
2A	nt	74.8	75.3–75.6	77.4–78.1	79.3–79.6	77.8	76.5–78.4	81.7–83.2	89.7–92.7	86.5–86.7	82.7–84.5	83.3–85.4
	aa	95.2	95.2–95.9	95.9–96.6	96.6	96.6	95.2–96.6	95.2–95.9	98.6	96.6–97.3	95.9–96.6	95.9
2B	nt	72.9	66.8–67.9	68.8–70.4	72.5	68.3	67.9–68.4	83.9–85.2	92.5–94.7	86.5	68.3–71.1	81.5–82.4
	aa	96.9	91.6–92.7	92.7	94.8	92.7	92.7	96.9	98.0–100	98.0	92.7–94.8	95.9–98.0
2C	nt	75.3	73.0–74.1	74.9–75.1	74.8–75.0	73.5–73.7	73.9–73.8	86.0–86.9	92.6–94.1	86.6–87.1	73.5–75.7	85.0–85.3
	aa	81.6	81.6–82.8	82.4–83.2	82.8–83.2	82	81.2–82.4	89.4–89.8	92.7–94.1	89.0–89.4	82.4–84.4	86.4–87.5
3A	nt	70.2	66.8	64.9–65.6	66.8–67.5	66.8–67.4	65.5–66.8	86.4–87.4	89.8–92.5	86.0–86.5	64.9–67.7	72.5–73.1
	aa	88.9	90.2	86.3–88.9	90.2	86.3–87.6	88.9	95.2	96.4	94.0	88.9–90.2	92.8–94.0
3B	nt	58.5	72.4	79.1–81.1	72.1	74.7	74.7–76.9	86.3–88.3	96.9–98.5	98.5	61.2–67.0	77.8–80.1
	aa	90.5	90.5	85.3–90.5	95.3	95.3	95.3	100.0	100.0	100.0	90.5	100.0
3C	nt	67.7	70.4–70.9	69.8–71.0	69.3–69.6	70.4–71.0	69.2–69.8	87.7–88.4	92.0–93.4	89.1–89.3	69.8–72.1	76.4–77.2
	aa	89.0	93.8	93.8	93.8	92.6	90.8–91.4	99.4	98.9–100	98.9–99.4	92.0–93.8	98.3–98.9
3D	nt	73.1	73.1–73.7	74.5–75.3	74.6–74.8	74.3–74.6	74.2–75.2	85.6–86.1	91.3–92.3	84.9–85.0	70.9–73.0	78.4–78.9
	aa	92.6	93.7–94.4	93.7–94.4	92.3–92.8	94.7	94.2–94.4	97.8	97.1–98.5	97.6	92.6–93.5	96.7
Genome	nt	75.2	75.8–76	76.2–76.6	76.3–76.5	75.6–75.7	76–76.2	86.3–86.4	91.0–93.3	87.9	79.2–79.5	83.1–83.3

^∗^*Subgenotype A: BrCr-CA-70 (GenBank accession no. U22521), B1: 236-TW86 (FJ357379) and 244-TW86 (FJ357381), B2: MS/7423/87(U22522) and 20233/1983(AB575923), B3: SAR/SHA66 (AM396586) and 26M/AUS/4/99 (EU364841), B4:5865/SIN/000009 (AF316321) and 5666/SIN/002209 (AF352027), B5: 2007-08747 (EU527985), 2009-03531 (HM622390) and S19841-SAR-03 (DQ341363), C1: 1M-AUS-12-00 (DQ341361) and 804/NO/03 (DQ452074), C2:1245a/98/TW (AF176044), ENT/PM/SHA71 (AM396585), Tainan/5746/98 (AF304457) and 6F/AUS/6/99 (DQ381846), C3: 03/KOR/00 (DQ341355) and06/KOR/00 (DQ341355), C4: SHZH03-CHN (AY465356), 1235/04/TW(DQ133459), BJ08/Z004/3 (FJ606447), 036/SH/CHN/2009 (FJ713137) and 1/SZ/08 (FJ607334), C5: 2007-07364 (EU527983), and E2005125-TW (EF063152)*.

### Neutralizing Antibody Against FY17 and SY32-10

**FIGURE 2:**
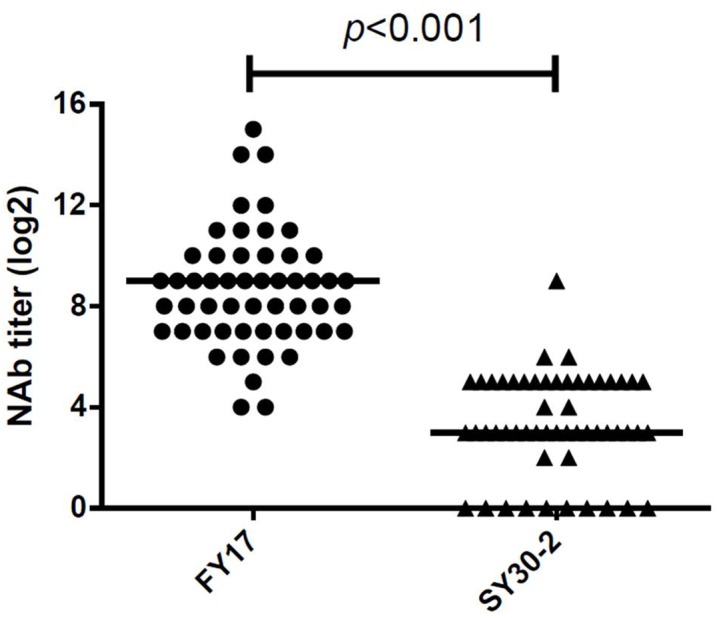
Responses of serum from subgenotype C4 of EV71 infected cases against FY17 and SY30-2. The two horizontal lines in this figure represent the median value of log2 transformed NTAb titer.

A total of 52 serum samples within 5 days of HFMD onset were collected from subgenotype C4 of EV71 infected children. All EV71 infected cases had detectable NTAb titers against FY17 and ranged from 16 to 32768 with the median NTAb titer of 512 [Interquartile range (IQR):128, 1024]. Cross-reactive NTAb titers against SY30-2 ranged from 0 to 256 with the median NTAb titer of 8 (IQR: 8, 32). There were no NTAbs against SY30-2 in 11 (20.4%) serum samples. Significant difference in the NTAb titers was found between FY17 and SY30-2 (*P* < 0.001) (**Figure 2**). The NTAb titers of all serum samples against FY17 were significantly higher than those against SY30-2. NTAb titers of serum samples against FY17 and SY30-2 were substantially correlated (*R*^2^ = 0.453, *P* = 0.001). Five serum samples from children without HFMD symptoms were collected as controls. Four children had no detectable NTAb titers against both FY17 and SY30-2. One serum sample had the NTAb titer of 2048 against FY17 and 32 against SY30-2, which we speculated having a latent infection.

Serum samples were divided into two groups based on whether there were NTAbs against SY30-2. In the group with positive NTAbs against SY30-2, the median NTAb titer against FY17 was 512 (IQR: 128, 1024), and it was significantly higher than that in the group with negative NTAbs against SY30-2(*P* = 0.049), in this group the median NTAb titer against FY17 was 192 (IQR: 28, 448).

### Different Amino Acid Residues on Subgenotypes C2 and C4 of EV71

Pairwise comparisons of VP1, VP2, VP3 and VP4 between FY17 and SY30-2 showed that the amino acid differences between FY17 and SY30-2 were 1.4, 0.0, 0.4, and 0.0%, respectively. Five amino acid residues were different with four residues in VP1 (R22H, D31N, K98E, I249V) and one residue in VP3 (S93N).

In order to find out if these five amino acid residues were highly conserved on different subgenotypes, 28 VP1 sequences and VP3 sequences from EV71 subgenotype C4 (including three strains of EV71 vaccines available in China) and 21 VP1 sequences and VP3 sequences from subgenotype C2 of EV71 were collected (Supplementary Table S1). Result showed that VP1-22H (100.0%), VP1-31N (100.0%), VP1-249V (100.0%) and VP3-93N (100.0%) were highly conserved on subgenotype C4 of EV71. Significant difference was found in the distribution of those amino acid residues between subgenotypes C4 and C2 (*p* < 0.05) (**Table 2**).

**Table 2:** Analysis of the frequencies of amino acid residue between subgenotypes C4 and C2 of EV71.

Protein	AA location	subgenotype C4 n (%)	subgenotype C2 n (%)	*P*	OR 95%CI
VP1	22/R	0(0.0)	18(85.7)	**<0.001**	**^a^30 (4.4–206.1)**
	22/H	29(100.0)	1(4.8)		
	22/Q	0(0.0)	2(9.5)		
	31/D	0(0.0)	7(33.3)	**0.001**	**3.1 (2.0–4.7)**
	31/N	29(100.0)	14(66.7)		
	98/K	3(10.3)	3(14.3)	0.677	^b^1.3 (0.5–3.1)
	98/E	26(89.7)	17(81.0)		
	98/G	0(0.0)	1(4.8)		
	249/I	0(0.0)	19(90.5)	**<0.001**	**15.5 (4.1–59.2)**
	249/V	29(100.0)	2(9.5)		
VP3	93/S	0(0.0)	15(93.8)	**<0.001**	**30(4.4–206.1)**
	93/N	29(100.0)	1(6.3)		

^a^*Group of 22/Q was excluded when OR was calculated. ^*b*^Group of 98/G was excluded when OR was calculated. The bold values means significant difference was obtained between compared groups*.

### 3D Structure of VP1 and VP3 and Properties of Different Amino Acid Residues

**FIGURE 3:**
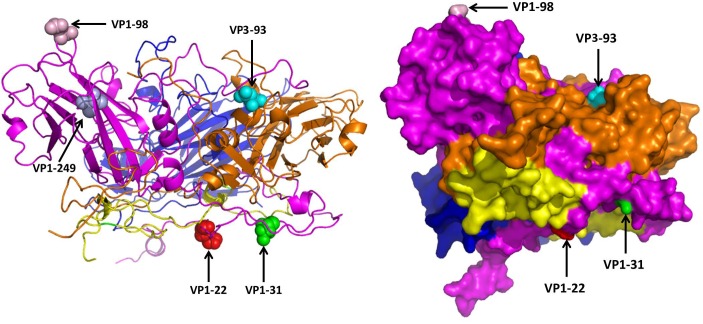
Five different amino acid residues on the viral capsid protein of FY17 and SY30-2 were labeled on 3D structure of EV71 capsid protein (PDB: 4cew) using Pymol software. VP1 (purple), VP2 (blue), VP3 (brown), VP4 (yellow). The red ball, green ball, pink ball and blue ball represent the 22 th, 31 th, 98 th, and 249 th amino acid residues on VP1. The cyan ball represents the 93 th amino acid residue on VP3.

The 3D structure of EV71 capsid (PDB: 4CEW) was obtained to locate the amino acid residues VP1-22, VP1-31, VP1-98, VP1-249, and VP3-93. As **Figure 3** shows, VP1-22, VP1-31, VP1-98 and VP3-93 were located on the random coil at the surface of viral capsid protein. VP1-249 was located on the sheet, which wasn’t on the surface of VP1. Among the different amino acid residues at the viral protein surface of SY30-2 and FY17, the VP1-22 and VP3-93 showed different hydropathy, the VP1-22, VP1-98 and VP3-93 showed different volume, the VP1-31, VP1-98 and VP3-93 showed different chemical characteristics. Amino acid properties including hydropathy, volume and chemical characteristics were all different for VP3-93 on SY30-2 and FY17 (**Table 3**).

**Table 3:** Analysis of the properties of amino acid residues that are different between SY20-2 and FY17.

Protein	Strain	AA location	Hydropathy	Volume	Chemical characteristics
VP1	SY30-2	22/R	Hydrophilic	Large	Basic
	FY17	22/H	Neutral	Medium	Basic
	SY30-2	31/D	Hydrophilic	Small	Acidic
	FY17	31/N	Hydrophilic	Small	Amide
	SY30-2	98/E	Hydrophilic	Medium	Acidic
	FY17	98/K	Hydrophilic	Large	Basic
	SY30-2	249/I	Hydrophobic	Large	Aliphatic
	FY17	249/V	Hydrophobic	Medium	Aliphatic
VP3	SY30-2	93/S	Neutral	Very small	Hydroxyl
	FY17	93/N	Hydrophilic	Small	Amide

## Discussion

Subgenotype C2 of EV71 has been widely detected in Australia, the Netherlands, Japan, Singapore and Thailand for several years (Huang et al., 2011; World Health Organization [WHO], 2011). However, C4 EV71 was the single predominant subgenotype circulating in the mainland of China since the first EV71 was isolated in China in 1998 (Yan et al., 2012). In this study, a subgenotype C2 of EV71 was isolated from a child who got HFMD 6 days after he went to Thailand. Since the common incubation period of HFMD ranges from three to 6 days (Hoy et al., 2012), and the subgenotype C2 of EV71 was not prevalent in China but common in Thailand, we speculated that this child got infected in Thailand.

To our knowledge, this was the first time a subgenotype C2 of EV71 from HFMD was isolated in Beijing, and it was the second time a subgenotype C2 of EV71 was isolated in China. The first isolate in China was reported in 2012. It was obtained from an AFP case in 1996 (Tao et al., 2012). These two C2 isolates in China showed high identity in VP1. Unlike the first subgenotype C2 of EV71 isolated in Korea in 2014 which was a recombinant strain (Lee et al., 2016), SY30-2 shared high genetic identity with C2 reference strains in each gene fragment. Owing to frequent international travel, there is a great possibility of importing other genotypes of EV71 into China. Accordingly, there is an urgent need to monitor the emergence of new viruses and recombination events to prevent HFMD outbreak among low age children.

In this study, we found serum from local people infected with C4 EV71 didn’t show high NTAb titer against SY30-2. This was inconsistent with a previous study, in which serum from C4 EV71-induced HFMD and subclinical patients all contained NTAbs against subgenotype C2 of EV71 strains (Mao et al., 2013). We speculated that the serum from the acute stage might contribute to low NTAb titer compared with serum from recovering period in the above study. Moreover, we found that NTAb titer against FY17 and SY30-2 were correlated. NTAb titers against FY17 in the group with positive antibody against SY30-2 were significantly higher than that in the group with no antibody against SY30-2. This result showed that the level of NTAb titer might be associated with the patient’s immune response.

In this study, we found that serum from subgenotype C4 of EV71 infected people could provide NTAbs against subgenotype C2 of EV71 infection. This result was in line with other studies (Huang et al., 2010). However, according to a previous study, although a C2 strain could be effectively neutralized, a C2-like strain which was genetically recombinant couldn’t be effectively neutralized by serum from C4 infected cases (Huang et al., 2013). Due to the continuous mutation of the virus itself and the selection pressure initiated by massive vaccination programs, novel strains of EV71 may emerge at any time. Therefore, systematic pathogenic surveillance and efficacy evaluation of the vaccine in use is highly required to get us prepared for any upcoming challenges.

In this study, significant difference was found in the NTAb titers against FY17 and SY30-2 which were likely due to the structure difference caused by four amino acid residues on VP1 and VP3. According to a previous study (Arthur Huang et al., 2017), potent and broadly reactive antibodies recognized neutralizing epitopes on the rims of the capsid canyon. In this study, VP1-98 was located on the canyon northern rim, and its mutation on SY30-2 might have led to the reduced NTAb titer. Moreover, since VP1-22, VP1-31, VP1-98 and VP3-93 were all located in the random coil at the surface of the virion, we assumed that they might play an important role in stimulating the body to produce high NTAb titer. However, reverse genetics would be required to identify molecular determinants of these three amino acid residues between subgenotype C2 and C4 of EV71.

## Conclusion

In conclusion, this is the first report of an EV71 subgenotype C2 isolated from HFMD in Beijing, China. Only a few antigenic variations on EV71 could lead to a great change in NTAb titer. We suggest that the epidemiology of new genotype and subgenotype of EV71 should be closely monitored and the efficacy of available vaccines against newly introduced viruses should be evaluated from time to time.

## Author Contributions

JL and YL carried out the experimental design, participated in the experiment, and drafted the manuscript. SZ, HM, and XL participated in the experimental design, performed the data analysis, and reviewed the manuscript. ZL, WZ, HJ, and DH participated in the data collection and analyzed and reviewed the manuscript. YD, YY, and LC participated in the experiment and reviewed the manuscript. QW contributed to the experimental design and provided a final review of this manuscript. All authors read and approved the final manuscript.

## Conflict of Interest Statement

The authors declare that the research was conducted in the absence of any commercial or financial relationships that could be construed as a potential conflict of interest.

## Supplementary Material

FIGURE S1 Similarity plot analysis of SY30-2 and C2 subgenotype EV71 strains (DQ381846, AF176044), B3 subgenotype EV71 reference strain (EU364841), and a C2-like strain (HM622391) on the basis of full-genomes. SY30-2 was used as the query sequence.

## Abbreviations

EV71: enterovirus 71
HFMD: hand foot and mouth disease
NTAb: neutralizing antibody
