# Supplementary figures and images for: Analysis of an Imported Subgenotype C2 Strain of Human Enterovirus 71 in Beijing, China, 2015
Source: Front Microbiol. 2018 Sep 28;9:2337. doi: 10.3389/fmicb.2018.02337 (PMC6172327; doi:10.3389/fmicb.2018.02337)

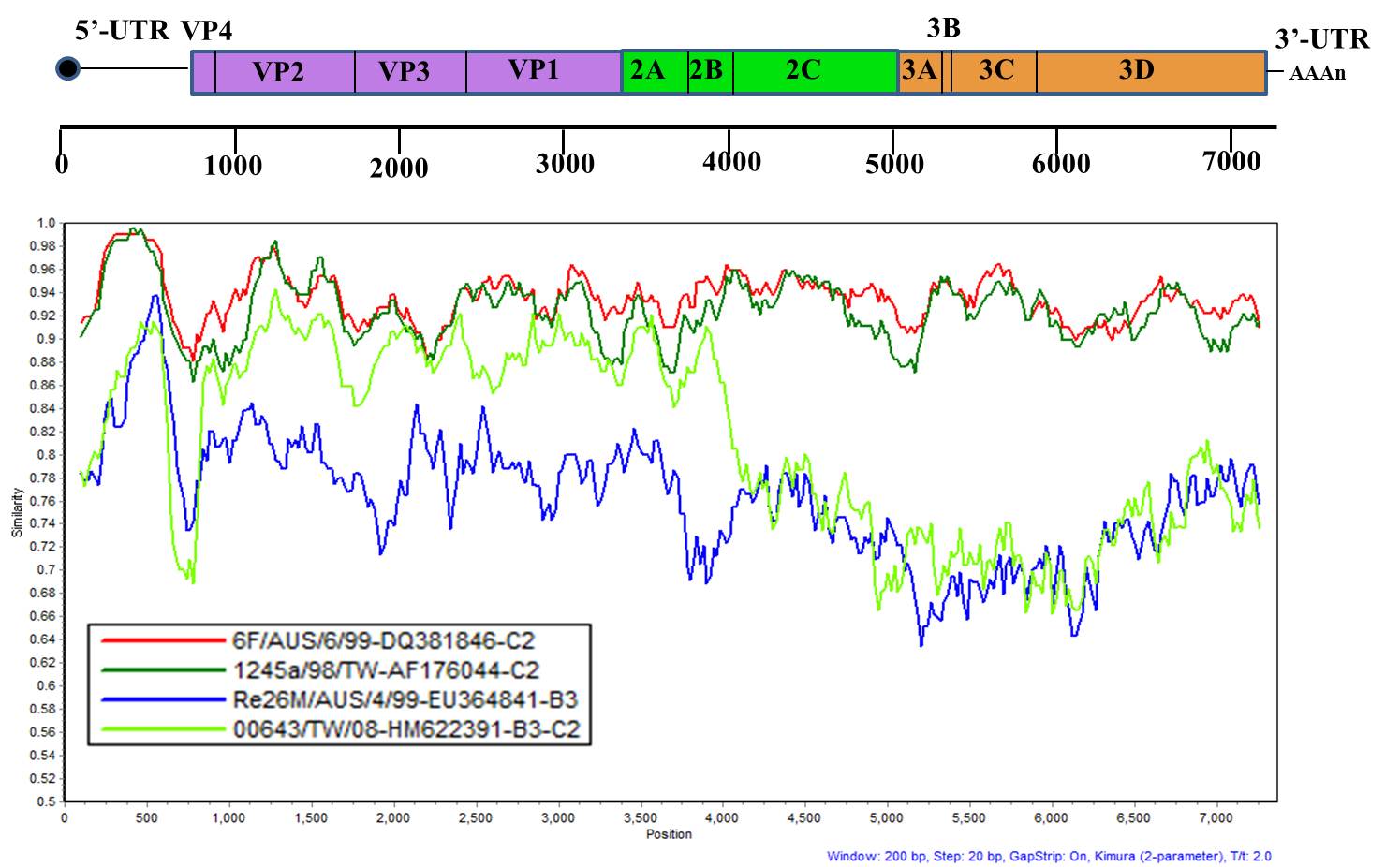

Supplement: FIGURE S1 — Similarity plot analysis of SY30-2 and C2 subgenotype EV71 strains (DQ381846, AF176044), B3 subgenotype EV71 reference strain (EU364841), and a C2-like strain (HM622391) on the basis of full-genomes. SY30-2 was used as the query sequence. [file Image_1.JPEG]
