# Supplementary material for: Analysis of an Imported Subgenotype C2 Strain of Human Enterovirus 71 in Beijing, China, 2015
Source: Front Microbiol. 2018 Sep 28;9:2337. doi: 10.3389/fmicb.2018.02337 (PMC6172327; doi:10.3389/fmicb.2018.02337)
Supplement: Supplementary file 2 [file Table_1.DOCX]

**TABLE S1 | EV71 reference strains enrolled in this study.**

| GenBank Accession number | Isolate | Genotype or Subgenotype |
| --- | --- | --- |
| U22521 | BrCr-CA-70 | A |
| FJ357379 | 236-TW86 | B1 |
| FJ357381 | 244-TW86 | B1 |
| AB575923 | 20233/1983 | B2 |
| U22522 | MS/7423/87 | B2 |
| AM396586 | SAR/SHA66 | B3 |
| EU364841 | 26M/AUS/4/99 | B3 |
| AF316321 | 5865/SIN/000009/2000 | B4 |
| AF352027 | 5666/SIN/002209/2000 | B4 |
| DQ341363 | S19841-SAR-03 | B5 |
| EU527985 | 2007-08747 | B5 |
| HM622390 | 2009-03531 | B5 |
| DQ341361 | 1M-AUS-12-00 | C1 |
| DQ452074 | 804/NO/03 | C1 |
| AB491220 | 265/Netherlands/2008 | C2 |
| AB524272 | 3890-99/Netherlands/1999 | C2 |
| AB524277 | 4359/Netherlands/2002 | C2 |
| AB524279 | 979/Netherlands/2000 | C2 |
| AB552985 | 365/Netherlands/2000 | C2 |
| AB552988 | 3692/Netherlands/200 | C2 |
| AF119796 | TW/2086/98 | C2 |
| AF135947 | 2641/AUS1995 | C2 |
| AF176044 | 1245a/98/TW | C2 |
| AF304457 | TW/5746/98 | C2 |
| AF376109 | 8M-6/AUS/1999 | C2 |
| AM396585 | ENT/PM/SHA71 | C2 |
| DQ381846 | 6F/AUS/6/99 | C2 |
| GU198370 | FY08-C30/CHN | C2 |
| JN544418 | 4643-TW98 | C2 |
| JN835312 | MRS/09/3663 | C2 |
| JN874554 | Tainan-5746-TW-98 | C2 |
| JQ621841 | 323/PHL/2002 | C2 |
| JQ621842 | 80/PHL/2005 | C2 |
| JX025559 | LAZ60-TR/Aus | C2 |
| KC436270 | V08-2236079/HK/CHN | C2 |
| KC589302 | BOR1718-FRA12 | C2 |
| KC589303 | LYO29244-FRA10 | C2 |
| KF306101 | NHRIEV95528-TW-Jun-25-12 | C2 |
| DQ341355 | 06-KOR-00 | C3 |
| DQ341356 | 03-KOR-00 | C3 |
| AY465356 | SHZH03/CHN | C4 |
| DQ133459 | 1235/TW/2004 | C4 |
| EU703812 | FY/17.08/1/CHN | C4 |
| EU703814 | FY/17.08/3/CHN | C4 |
| EU812515 | FY23/KM/CHN | C4 |
| FJ439769 | FY0805/CHN | C4 |
| FJ606447 | BJ08-Z004-3 | C4 |
| FJ607334 | 01-SHENZHEN/08/CHN | C4 |
| FJ607336 | 28/SHENZHEN/08/China/HFMD/2008 | C4 |
| FJ713137 | 036/SH/CHN/2009 | C4 |
| FJ828519 | BJ2008/CHN | C4 |
| GQ994991 | CQ3-09-CHN | C4 |
| GQ994992 | HN2-09/CHN | C4 |
| GU198367 | 1FY08-C30-P2/CHN | C4 |
| HQ712020 | MP10/BJ/CHN/2010 | C4 |
| JN256059 | G288-927F/HeN/CHN/2009 | C4 |
| JX025561 | FY7VP5/AH/CHN/2008 | C4 |
| JX025561 | FY7VP5/AH/CHN/2008 | C4 |
| JX244182 | 2009SDLY1/CHN | C4 |
| JX678875 | FY17.08-4/AH/CHN/2008 | C4 |
| KF444809 | Queenmary/HongKong/2012 | C4 |
| KJ004552 | Hun11-32/CHN/2011 | C4 |
| KJ004553 | Hun11-4/CHN/2011 | C4 |
| KJ004555 | Hun12-10/CHN/2012 | C4 |
| KJ004560 | GD10-45/CHN/2010 | C4 |
| KP308411 | KHM/351/2012 | C4 |
| KP308459 | KHM/336/2012 | C4 |
| KT428644 | SZ04/CHN/2014 | C4 |
| KX197461 | SEP001/Cambodia/2012 | C4 |
| EF063152 | E2005125-TW | C5 |
| EU527983 | 2007-07364/TW | C5 |
